# Omission of Contralateral Systematic Biopsies in Unilateral Suspicious Prostate Cancer on Magnetic Resonance Imaging: Implications for Radiation Treatment Selection

**DOI:** 10.1016/j.euros.2025.01.006

**Published:** 2025-01-24

**Authors:** Daniël L. van den Kroonenberg, Sanne J. Jonker, Auke Jager, Joëlle D. Stoter, Eva Schaake, Karel A. Hinnen, Wietse S.C. Eppinga, Ivo G. Schoots, Jochem R.N. van der Voort van Zyp, André N. Vis

**Affiliations:** a Department of Urology Amsterdam UMC location VUmc Amsterdam The Netherlands; b Department of Radiotherapy Netherlands Cancer Institute – Antoni van Leeuwenhoek Hospital Amsterdam The Netherlands; c Department of Radiotherapy Amsterdam UMC Amsterdam The Netherlands; d Department of Radiotherapy UMC Utrecht Utrecht The Netherlands; e Department of Radiology and Nuclear medicine Erasmus MC Rotterdam The Netherlands; f Department of Radiology Netherlands Cancer Institute – Antoni van Leeuwenhoek Hospital Amsterdam The Netherlands; g Prostate Cancer Network Netherlands Amsterdam The Netherlands

**Keywords:** Prostate cancer, Unilateral lesion, Perilesional biopsy, Prostate biopsy, Systematic biopsy, Targeted biopsy, Radiation therapy, Hormone therapy, Magnetic resonance imaging

## Abstract

**Background and objective:**

A combined approach of magnetic resonance imaging (MRI) targeted biopsies (TBx) and systematic biopsies (SBx) was recommended previously in patients with unilateral suspicious prostate cancer (PCa) on MRI. Yet, new PCa guidelines suggest that contralateral SBx can be omitted. It is unknown how this guideline modification impacts treatment selection. This study evaluates the value of contralateral SBx in radiation treatment selection in patients with unilateral suspicious lesions (Prostate Imaging Reporting and Data System [PI-RADS] ≥3) on MRI.

**Methods:**

Case files of 80 patients with a unilateral suspicious lesion on diagnostic MRI who underwent TBx and bilateral SBx were collected. The cases were presented to four radiation oncologists twice: once with diagnostic information of bilateral SBx and TBx, and once with diagnostic information of ipsilateral SBx and TBx pathology results. Based on this information, external beam radiation treatment (EBRT) fractionation scheme, duration of androgen deprivation therapy (ADT), and feasibility of brachytherapy (monotherapy or brachyboost) were considered.

**Key findings and limitations:**

After omitting information of contralateral SBx pathology results, selection of EBRT fractionation scheme and ADT duration changed in 14% (95% confidence interval [CI] 9.8–17) and 15% (95% CI 11–19) of cases, respectively. The feasibility of brachytherapy as monotherapy and brachyboost, respectively, changed in 11% (95% CI 7.9–15) and in 21% (95% CI 17–26) of cases, with overall poor interobserver variability for both diagnostic scenarios (Fleiss’ kappa 0.15 and 0.16).

**Conclusions and clinical implications:**

Our findings indicate that omitting contralateral SBx has an impact on the treatment selection of patients who choose for radiation therapy as their treatment for locally confined PCa.

**Patient summary:**

In patients with prostate cancer identified via magnetic resonance imaging on one side of the prostate, exclusion of prostate biopsies from the opposite side affected the selection of radiation treatment.

## Take Home Message

Diagnostic information from contralateral systematic biopsies has a notable impact on radiation fractionation, androgen deprivation therapy duration, and brachytherapy use in patients with unilateral magnetic resonance imaging lesions, with high-dose rate boost being most affected and substantial interobserver variability observed.

## 1 Introduction

Today, prostate cancer (PCa) ranks as the second most prevalent cancer in men [1]. The European Association of Urology (EAU) recommends utilizing magnetic resonance imaging (MRI) in cases of suspected PCa marked by elevated levels of prostate-specific antigen (PSA) or suspicious findings on digital rectal examination (DRE). Subsequently, MRI-targeted (TBx) and bilateral systematic (SBx) prostate biopsies were recommended as the diagnostic strategy to prove or rule out PCa. However, the value of SBx in patients with unilateral suspicious MRI lesions (Prostate Imaging Reporting and Data System [PI-RADS] ≥3) has been called into question [2]. A meta-analysis comprising eight retrospective studies has suggested that TBx combined with perilesional biopsies has comparable detection rates for clinically significant PCa (csPCa) to TBx and bilateral SBx [3]. This evidence has recently led to an EAU guideline modification, recommending perilesional biopsies over bilateral SBx in patients with a unilateral MRI lesion [4].

The revision in the diagnostic approach prompts questions into whether the absence of information gained from (contralateral) SBx impacts treatment selection and oncological outcomes. In previous studies, the diagnostic information of contralateral SBx has demonstrated limited value in aiding urologists in their treatment planning of robot-assisted radical prostatectomy (RARP) in patients with a unilateral suspicious MRI lesion [5]. However, its additional value in guiding radiation treatment options remains unknown.

Radiation options for PCa treatment include external beam radiation therapy (EBRT), possibly combined with high-dose rate (HDR) brachytherapy boost and/or hormone therapy (HT) such as androgen deprivation therapy (ADT) [2]. RARP and radiation treatment have similar oncological outcomes in localized PCa, making radiation treatment a viable alternative for patients who are not suitable candidates for surgery [6]. Radiation therapy tends to have fewer short-term side effects, particularly regarding erectile dysfunction and urinary incontinence, than RARP [7]. Efficacy of the radiation treatment relies on the assessment of tumor characteristics and is reviewed thoroughly before treatment [8].

The aim of this study was to investigate the additional value of the diagnostic information gained by contralateral SBx in the context of radiation treatment selection for PCa in patients with a unilateral suspicious MRI lesion.

## 2 Patients and methods

This study was ethically approved by the Medical Research Ethics Committee Academic Medical Centre Amsterdam (MREC AMC, ID: W21_534 #21.590). Patient data were collected as part of an earlier study to determine the impact of omitting contralateral SBx on the surgical planning of patients undergoing RARP [5].

### 2.1 Study population

A total of 80 consecutive patients with biopsy-proven PCa, diagnosed between 2018 and 2023, were included (Table 1). The inclusion criteria were patients with a unilateral MRI lesion (PI-RADS ≥3) who underwent both TBx and bilateral SBx. PCa was defined as International Society of Urological Pathology (ISUP) grade group ≥2 [9].

**Table 1:** Patient characteristics

	*n* = 80		*p* value
Prebiopsy serum PSA (ng/ml), median (IQR)	6.9 (5.2–10)		
PSA density, median (IQR)	0.17 (0.12–0.30)		
Digital rectal exam, *N* (%)			
Benign	34 (43)		
Malignant	38 (48)		
Dubious	6 (7.5)		
Missing	2 (2.5)		
MRI result, *N* (%)			
PI-RADS 3	7 (8.8)		
PI-RADS 4	35 (44)		
PI-RADS 5	37 (46)		
Radiological tumor stage, *N* (%)			
mT2	58 (73)		
mT3a	18 (23)		
mT3b	4 (5.0)		
Pathology results	Bilateral SBx + TBx	Ipsilateral SBx + TBx	
Highest ISUP, *N* (%)			0.5
ISUP 2	35 (48)	37 (46)	
ISUP 3	23 (29)	21 (26)	
ISUP 4	6 (7.5)	6 (7.5)	
ISUP 5	16 (20)	16 (20)	
Positive biopsy cores, mean (% of total biopsies)	7 (47)	6 (67)	<0.01
Presence of cribriform growth, *N* (%)			
Yes	29 (36)		
No	18 (23)		
Missing	33 (41)		

IQR = interquartile range; ISUP = International Society of Urological Pathology; MRI = magnetic resonance imaging; PI-RADS = Prostate Imaging Reporting and Data System; PSA = prostate-specific antigen; SBx = systematic biopsies; TBx = target biopsies.

### 2.2 Study design

Four experienced radiation oncologists from three academic centers chose a radiation treatment scheme using the diagnostic data from 80 patients on two different occasions. The cases were randomized to ensure that the radiation oncologists were blinded to the outcomes of previous cases. One round involved the diagnostic information gained by both TBx and bilateral SBx pathology results, while the other included TBx and ipsilateral SBx pathology results only. Besides pathology results, data included PSA, PSA density, DRE, and MRI results (prostate volume, lesion location, PI-RADS, and mT stage). The radiation treatment scheme was presented via a multiple-choice questionnaire. There were four queries regarding EBRT fractionation: (1) ultrahypofractionated scheme: 5 × 7–7.25 Gy; (2) moderately hypofractionated scheme: 20 × 3–3.1 Gy; and (3) conventional fractionated scheme: 35–39 × radiobiologically >76 Gy, HT/ADT (ie, [1] none, [2] 6 mo, or [3] 18–36 mo), brachytherapy applicable (yes/no), and HDR boost with EBRT applicable (yes/no) [10]. Radiation oncologists were allowed to give only a single answer per query. A total of 640 cases were retrieved over a 7-mo period, with each radiation oncologist contributing 160 cases (2 × 80).

### 2.3 MRI protocol

MR image acquisition and reporting adhered to the PI-RADS guidelines and were performed by uroradiologists [11]. MRI sequences comprised T1-weighted, T2-weighted, and diffusion-weighted imaging, and apparent diffusion coefficient maps (1.5 or 3 Tesla). MRI reports were retrieved from the electronic patient records and constituted part of the standard of care. No revisions were conducted for the purposes of this study.

### 2.4 Biopsy protocol and histopathological assessment

Experienced physicians conducted transrectal prostate biopsy until July 2020, thereafter transitioning to the transperineal approach. Transrectal prostate biopsy employed the Philips iU-22 ultrasound system with an end-firing probe. Biopsy typically involved a 12-core SBx and two- to three-core TBx per MRI lesion, aided by the MRI/ultrasound fusion software of ProFuse combined with the Artemis fusion system.

Transperineal prostate biopsy utilized the BK5000 ultrasound system with a biplane probe. The probe was stabilized on a platform, and biopsy was conducted using a brachytherapy template grid. Biopsy typically comprised 14-core SBx and two- to three-core TBx per MRI lesion, utilizing MIM fusion software.

Biopsy cores underwent evaluation by an experienced uropathologist following ISUP guidelines [9].

### 2.5 Statistical analysis

SPSS was utilized for descriptive statistical analysis. McNemar (Bowker) test was employed to compare the paired outcomes of the radiation treatment schemes. Cohen's kappa was employed for intraobserver agreement and Fleiss' kappa for interobserver agreement. The kappa values were categorized as poor agreement (0.00–0.40), fair agreement (0.40–0.75), or excellent agreement (0.75–1.00) [12].

## 3 Results

### 3.1 Clinicopathological and radiological characteristics

Patient characteristics, imaging outcomes, and biopsy results are presented in Table 1. The median serum PSA level was 6.9 ng/ml (interquartile range [IQR] 5.2–10) with a median PSA density of 0.17 (IQR 0.12–0.30).

MRI results showed that 8.8% of patients had PI-RADS 3, 44% had PI-RADS 4, and 46% had PI-RADS 5. Regarding mT stage, 73% were staged as mT2, 23% as mT3a, and 5% as mT3b.

Pathology results indicated that ISUP 2 was found in 48% of patients in the bilateral SBx + TBx scenario and in 46% in the ipsilateral SBx + TBx scenario. Consequently, in two cases, contralateral SBx resulted in the identification of a higher-grade tumor from ISUP 2 to ISUP 3. ISUP 3 was found in 29% of the bilateral SBx + TBx scenario and 26% of the ipsilateral SBx + TBx scenario. ISUP 4 was present in 7.5% of both scenarios, and ISUP 5 was present in 20% of both scenarios. The mean numbers of positive cores were 7 (47%) in the bilateral SBx + TBx scenario and 6 (67%) in the ipsilateral SBx + TBx scenario.

### 3.2 External beam radiation therapy

Using the diagnostic information gained from bilateral SBx + TBx (*n* = 80 × 4 readers) versus ipsilateral SBx + TBx (*n* = 80 × 4 readers), the ultrahypofractionated scheme was selected as the preferred treatment in 163/320 (51%) versus 175/320 (55%) cases (difference 3.8%, 95% confidence interval [CI] 1.1–4.0), moderately-hypofractionation scheme in 147/320 (46%) versus 135/320 (42%) cases (difference 3.8%, 95% CI –3.9 to 11), and normal fractionated scheme in 7/320 (3%) versus 9/320 cases (difference 0.6%, 95% CI –3.0 to 1.8), with a *p* value of 0.18.

After omitting contralateral SBx, 43 cases (14%; 95% CI 9.8–17) changed treatment selection, with treatment intensification occurring in 18 cases and treatment reduction in 25 cases. The interobserver agreement for radiation oncologists was fair for both the bilateral SBx + TBx and the ipsilateral SBx + TBx scenario, with Fleiss’ kappa values of 0.55 and 0.53, respectively. Intraobserver agreement ranged from fair to excellent, with Cohen’s kappa varying from 0.42 to 0.79 (Table 2).

**Table 2:** External beam radiation therapy fractionation scheme

	Ultrahypofractionated scheme		Moderately hypofractionation scheme		Normal fractionated scheme		
	Bilateral SBx + TBx	Ipsilateral SBx + TBx	Bilateral SBx + TBx	Ipsilateral SBx + TBx	Bilateral SBx + TBx	Ipsilateral SBx + TBx	Cohen’s kappa
R1 a	47 (59%)	51 (61%)	32 (40%)	29 (36%)	0	0	0.77
R2	49 (61%)	52 (65%)	30 (38%)	27 (34%)	1 (1.3%)	1 (1.3%)	0.79
R3	24 (30%)	26 (23%)	49 (61%)	46 (58%)	7 (9%)	8 (10%)	0.42
R4 a	43 (54%)	46 (58%)	36 (45%)	33 (41%)	0	1 (1.3%)	0.68
Total *n* (%, 95% CI)	163 (51%, 46–57)	175 (55%, 49–60)	147 (46%, 40–52)	135 (42%, 37–48)	7 (2.2%, 0.59–3.8)	9 (2.8%, 1.0–4.6)	
Fleiss’ kappa	0.55	0.53	0.55	0.53	0.55	0.53	

CI = confidence interval; R = Radiation oncologist, ultrahypofractionated scheme: 5 × 7–7.25 Gy, moderately hypofractionation scheme: 20 × 3–3.1 Gy, normal fractionated scheme: 35–39 × radiobiologically >76 Gy; SBx = systematic biopsies; TBx = target biopsies.

a One missing case.

### 3.3 Androgen deprivation therapy

Using the diagnostic information gained from bilateral SBx + TBx (*n* = 80 × 4 readers) versus ipsilateral lateral SBx + TBx (*n* = 80 × 4 readers), no ADT was recommended by the radiation oncologists in 163/320 (51%) versus 175/320 (55%) cases (difference 3.9%, 95% CI –1.1 to 4.0), 6 mo of ADT in 61/320 (19%) versus 54/320 (17%) cases (difference 2.1%, 95% CI –3.8 to 8.2), and 18–36 mo of ADT in 95/320 (30%) versus 91/320 (28%) cases (difference 1.3%, 95% CI –5.8 to 8.3; *p* = 0.05).

In 49 cases (15%; 95% CI 11–19), ADT duration altered as a result of the diagnostic information gained by contralateral SBx. Of these cases, 20 involved an increase in ADT duration and 29 involved a reduction of ADT duration. The interobserver agreement was fair for both scenarios, with Fleiss’ kappa values of 0.66 and 0.57, respectively. Intraobserver agreement ranged from fair to excellent, with Cohen’s kappa’s ranging from 0.68 to 0.85 (Table 3).

**Table 3:** Hormone therapy duration

	None		6 mo		18–36 mo		
	Bilateral SBx + TBx	Ipsilateral SBx + TBx	Bilateral SBx + TBx	Ipsilateral SBx + TBx	Bilateral SBx + TBx	Ipsilateral SBx + TBx	Cohen’s kappa
R1 a	31 (39%)	34 (43%)	19 (24%)	18 (23%)	29 (36%)	28 (35%)	0.71
R2	49 (61%)	54 (68%)	10 (13%)	7 (8.8%)	21 (26%)	19 (24%)	0.71
R3	38 (48%)	39 (49%)	18 (23%)	16 (20%)	24 (30%)	25 (31%)	0.68
R4	45 (56%)	48 (60%)	14 (18%)	13 (16%)	21 (26%)	19 (24%)	0.85
Total *n* (%, 95% CI)	163 (51%, 46–57)	175 (55%, 49–60)	61 (19%, 15–23)	54 (17%, 13–21)	95 (30%, 25–35)	91 (28%, 23–33)	
Fleiss’ kappa	0.66	0.57	0.66	0.57	0.66	0.57	

CI = confidence interval; R = Radiation oncologist; SBx = systematic biopsies; TBx = target biopsies.

a One missing case.

### 3.4 Applicability of brachytherapy

In the bilateral SBx + TBx setting, brachytherapy was chosen as a treatment alternative in 147 cases (44%). In the ipsilateral SBx + TBx setting, brachytherapy was a treatment alternative in 149 cases (45%; *p* = 0.87). After omitting contralateral SBx biopsy results, 36 cases (11%; 95% CI 7.8–15) received different indication for brachytherapy (21 cases from yes to no; 15 cases from no to yes). The interobserver agreement was fair for both settings, with kappa values of 0.63 and 0.59. Intraobserver agreement ranged from fair to excellent, with kappa values ranging from 0.58 to 0.82 (Table 4).

**Table 4:** Brachytherapy option

	Brachytherapy option		Change when leaving out contralateral biopsy results		Difference	Cohen’s kappa
	With result from contralateral SBx	Without result from contralateral SBx	Yes to no	No to yes		
R1 a	35 (44%)	35 (44%)	3 (3.6%)	3 (3.6%)	6 (7.2%)	0.80
R2	50 (63%)	52 (65%)	5 (6%)	3 (3.6%)	8 (9.6%)	0.78
R3 a	29 (36%)	31 (39%)	4 (4.8%)	2 (2.4%)	6 (7.2%)	0.82
R4	33 (41%)	31 (39%)	9 (11%)	7 (8.4%)	16 (19%)	0.58
Total *n* (%, 95% CI)	147 (46%, 41–52)	149 (47%, 41–52)	21 (6.6%, 3.9–9.3)	15 (4.7%, 2.3–7.7)	36 (11%, 7.9–15)	
Fleiss’ kappa	0.63	0.59				

CI = confidence interval; R = Radiation oncologist; SBx = systematic biopsies; TBx = target biopsies.

a One missing case.

### 3.5 HDR boost

In the bilateral SBx + TBx scenario, HDR boost with EBRT was an option in 168 cases (51%). In the ipsilateral SBx + TBx scenario, this was preferred in 151 cases (46%; *p* = 0.049). A total of 67 cases (21%, 95% CI 17–26) changed after omitting the contralateral SBx, with 37 cases switching from yes to no and 30 cases switching from no to yes. Interobserver agreement was poor for both scenarios, with kappa values of 0.15 and 0.16. Intraobserver agreement varied, with one radiation oncologist showing poor agreement (kappa value –0.03), while the other three showed fair to excellent agreement, with kappa values of 0.60–0.79 (Table 5).

**Table 5:** High-dose rate (HDR) boost option with external beam radiation therapy (EBRT)

	HDR boost with EBRT		Change when leaving out contralateral biopsy results		Difference	Cohen’s kappa
	With result from contralateral SBx	Without result from contralateral SBx	Yes to no	No to yes		
R1 a	61 (76%)	56 (68%)	13 (16%)	18 (23%)	31 (38%)	–0.03
R2 a	31 (39%)	28 (35%)	5 (6.3%)	2 (2.5%)	7 (8.9%)	0.79
R3	39 (49%)	35 (44%)	10 (13%)	6 (7.5%)	16 (20%)	0.60
R4	37 (46%)	32 (40%)	9 (11%)	4 (5.0%)	13 (16%)	0.67
Total *n* (%, 95% CI)	168 (53%, 47–58)	151 (47%, 42–53)	37 (12%, 8.1–15)	30 (9.5%, 6.2–13)	67 (21%, 17–26)	
Fleiss’ kappa	0.15	0.16				

CI = confidence interval; R = Radiation oncologist; SBx = systematic biopsies; TBx = target biopsies.

a One missing case.

## 4 Discussion

In 2024, the EAU guidelines modified the recommendations for diagnostic prostate biopsies for PCa [4]. As of today, perilesional prostate biopsies in combination with TBx instead of bilateral SBx combined with TBx are advised in those with an elevated PSA level, and a suspicion of PCa on DRE or diagnostic MRI (not recommended in cT3–4). This new approach has similar detection rates for csPCa to traditional SBx and TBx combined in patients with a unilateral MRI lesion [3]. However, it is unknown what consequences this change of diagnostic approach has with respect to different treatments strategies and oncological outcomes.

The aim of this study was to investigate the additional value of the diagnostic information gained by contralateral SBx in the context of radiation treatment options for PCa in patients with a unilateral MRI lesion. Our findings suggest that omitting contralateral SBx in patients with a unilateral lesion on diagnostic MRI has an observable impact on the radiation treatment selection. In approximately 10–20% of cases, the treatment scheme changed as a result of the information gained by contralateral SBx compared with TBx and ipsilateral SBx only.

An impact of the diagnostic information of contralateral SBx on the EBRT fractionation scheme decision was observed, but it was not significant (*p* = 0.18). Most differences were observed for the ultrahypofractionated and moderately hypofractionation schemes. Our findings indicated that in 14% (95% CI 9.8–17) of patients, fractionation scheme decision changed when contralateral SBx were not available. Although this appears to be a substantial difference, studies have demonstrated that these two radiation fractionation schemes can be equally oncologically effective for intermediate PCa [13], [14]. Consequently, the impact of this change in treatment may be limited and may be chosen for other reasons such as micturition problems or claustrophobia. Furthermore, it should be noted that the queries in our study do not fully reflect everyday clinical practice, where such decisions are more individualized and based on patient preference. Additionally, the radiation oncologists may have been influenced by the (in)availability of treatment options at their respective institutions, which could have affected their treatment choices.

With regard to the duration of ADT, omitting the diagnostic information of contralateral SBx led to a change in treatment plan in 15% (95% CI 11–19) of cases. It needs to be mentioned that some patients were advised a longer duration of ADT, whereas other were advised a shorter course of ADT. It is yet unknown what is the resulting oncological benefit or disadvantage of this lack of diagnostic information. In other words, it is unclear which subgroup of patients may benefit from this novel diagnostic approach of PCa. Additionally, a new national ADT protocol was accepted during the study, which may explain the difference in treatment scheme choices.

In 11% (95% CI 7.9–15) of cases, the treatment option of brachytherapy altered when the diagnostic information of contralateral SBx results was available. In a small proportion of patients, brachytherapy ceased to be a treatment option when contralateral biopsy pathology was present. This may be attributed to the fact that the contralateral SBx resulted in the tumor being categorized as high-risk rather than intermediate-risk PCa.

The selection of HDR boost with EBRT changed in 21% (95% CI 17–26) of cases when contralateral SBx results were available to the radiation oncologist (*p* = 0.49). This may be due to these pathology results indicating a more aggressive tumor than the ipsilateral SBx and TBx results alone. The impact of this change should not be underestimated. HDR boost with EBRT showed significantly higher biochemical disease-free survival than EBRT alone in 5 yr of follow-up [15]. This 21% change in HDR-boost application could therefore potentially result in an undertreatment in patients with unilateral lesions on MRI and not undergoing bilateral SBx. Furthermore, there was a considerable degree of interobserver variability among radiation oncologists regarding the selection of HDR boost in combination with EBRT. This variability may be attributed to the existence of disparate guidelines across institutions or a lack of equipment. Additionally, the absence of established guidelines pertaining to the administration of HDR boost may also be a contributing factor.

It should be noted that this study is not without limitations. The majority of the limitations identified are consistent with those observed previously in our study investigating the influence of contralateral SBx on the treatment planning of RARP [5]. For instance, the results are based on hypothetical treatment planning rather than actual treatment. Determining the actual impact of omitting contralateral SBx on the outcomes of radiation treatment options requires a prospective study. In addition, not all radiation treatment options were included in the questionnaire, potentially limiting the generalizability of our findings. Furthermore, the MRI results were not reviewed centrally. The differences in the diagnostic performance of MRI between observers and different centers may have impacted the outcomes of this study [16], [17]. Nevertheless, the treatment and radiation therapy schemes for both diagnostic scenarios were derived from identical case files and MRI scans. At last, this study examines ipsilateral SBx, which are not the same as perilesional biopsies. Perilesional biopsies are the recommended approach and focus on prostate tissue surrounding an MRI lesion. Ipsilateral SBx are taken from one half of the prostate. While these approaches are similar, their differences may have influenced the study results.

A noticeable limitation of this study is the absence of data on oncological and functional outcomes. While a change in biopsy approach may influence treatment strategy, it is unclear whether this translates to long-term changes in oncological and functional outcomes. This is an area that future research should address.

## 5 Conclusions

Omitting contralateral SBx in patients with a unilateral suspicious MRI lesion has an impact on the radiation fractioning schemes, ADT duration, and option of brachytherapy. Treatment selection of HDR boost with EBRT seems to be most affected by the omission of contralateral biopsies, although with substantial interobserver disagreement. Radiation oncologists should be aware that the omission of contralateral SBx may lead to different treatment decisions as compared with those in whom the diagnostic information of contralateral SBx is available. The impact of different radiation treatment strategies that are a result of different prostate biopsy schemes on the oncological outcome is yet unknown.

  ***Author contributions*:** Daniël L. van den Kroonenberg had full access to all the data in the study and takes responsibility for the integrity of the data and the accuracy of the data analysis.

  *Study concept and design*: van den Kroonenberg, Jonker, Jager, Stoter, Schaake, Hinnen, Eppinga, Schoots, van der Voort van Zyp, Vis.

*Acquisition of data*: van den Kroonenberg, Jonker, Stoter.

*Analysis and interpretation of data*: van den Kroonenberg, Jonker.

*Drafting of the manuscript*: van den Kroonenberg, Jonker.

*Critical revision of the manuscript for important intellectual content*: van den Kroonenberg, Jonker, Jager, Stoter, Schaake, Hinnen, Eppinga, Schoots, van der Voort van Zyp, Vis.

*Statistical analysis*: van den Kroonenberg, Jonker.

*Obtaining funding*: None.

*Administrative, technical, or material support*: None.

*Supervision*: van der Voort van Zyp, Vis.

*Other*: None.

  ***Financial disclosures:*** Daniël L. van den Kroonenberg certifies that all conflicts of interest, including specific financial interests and relationships and affiliations relevant to the subject matter or materials discussed in the manuscript (eg, employment/affiliation, grants or funding, consultancies, honoraria, stock ownership or options, expert testimony, royalties, or patents filed, received, or pending), are the following: None.

  ***Funding/Support and role of the sponsor*:** None.
